# SUV420H1 enhances the phosphorylation and transcription of ERK1 in cancer cells

**DOI:** 10.18632/oncotarget.6351

**Published:** 2015-11-19

**Authors:** Theodore Vougiouklakis, Kenbun Sone, Vassiliki Saloura, Hyun-Soo Cho, Takehiro Suzuki, Naoshi Dohmae, Houda Alachkar, Yusuke Nakamura, Ryuji Hamamoto

**Affiliations:** ^1^ Section of Hematology/Oncology, Department of Medicine, The University of Chicago, Chicago, IL 60637, USA; ^2^ Biomolecular Characterization Unit, RIKEN Center for Sustainable Resource Science, Wako, Saitama 351-0198, Japan

**Keywords:** ERK1, SUV420H1, protein lysine methyltransferase, non-histone protein methylation

## Abstract

The oncogenic protein ERK, a member of the extracellular signal-regulated kinase (ERK) cascade, is a well characterized signaling molecule involved in tumorigenesis. The ERK signaling pathway is activated in a large proportion of cancers and plays a critical role in tumor development. Functional regulation by phosphorylation of kinases in the ERK pathway has been extensively studied, however methylation of the ERK protein has not been reported to date. Here, we demonstrated that the protein lysine methyltransferase SUV420H1 tri-methylated ERK1 at lysines 302 and 361, and that substitution of methylation sites diminished phosphorylation levels of ERK1. Concordantly, knockdown of SUV420H1 reduced phosphorylated ERK1 and total ERK1 proteins, and interestingly suppressed ERK1 at the transcriptional level. Our results indicate that overexpression of SUV420H1 may result in activation of the ERK signaling pathway through enhancement of ERK phosphorylation and transcription, thereby providing new insights in the regulation of the ERK cascade in human cancer.

## INTRODUCTION

The extracellular signal-regulated kinase (ERK) cascade regulates numerous cellular processes, such as cell proliferation, differentiation and survival, by relaying extracellular signals and transmitting them throughout the cell [1–3]. This hierarchical cascade is comprised of many protein kinases, including Ras, Raf and MEK, whose sequential activation leads to the activation of ERK. The genes involved in this pathway are often subjects to mutations, as seen in the case of *Ras* and *Raf* genes in 30% and 7% of human cancers respectively, resulting in aberrant activation and deregulation of the ERK pathway [4, 5].

Two kinases, ERK1 (p44^MAPK^) and ERK2 (p42^MAPK^), are highly similar proteins with an amino acid homology of 84% [6]. Owing to this high similarity, their biological functions and regulatory mechanisms are also analogous, and their activation is induced by the same stimuli [7–9]. Post-translational modifications are known to play a fundamental role in the activation of the ERK kinases. Phosphorylation is essential for the nuclear translocation and activation of ERK1/2, which subsequently transduces downstream phosphorylation to various substrates. MEK-induced phosphorylation of the TEY domain of ERK1/2 causes a major conformational change and leads to detachment of ERK1/2 from scaffold proteins and other cytoplasmic anchors, making ERK1/2 accessible to Importin 7 (IPO7) for nuclear entry [10, 11]. Blockade of the IPO7 and ERK1/2 interaction has been shown to induce apoptosis of B-Raf melanoma cells, providing evidence that abrogation of ERK1/2 nuclear entry may inhibit proliferation [12]. ERK1/2 activates a wide array of nuclear substrates, not only by phosphorylation but also via direct protein-protein interactions, as seen in the case of Poly [ADP-ribose] polymerase 1 (PARP1) [13]. Recent findings show that Peptidylprolyl Cis/Trans Isomerase, NIMA-Interacting 1 (PIN1) overexpression and subsequent upregulation of RAB2A transcription in breast cancer stem-like cells results in abrogation of ERK1/2 dephosphorylation and inactivation by Dual Specificity Phosphatase 6 (DUSP6), which promotes tumorigenesis [14]. Furthermore, in K-RAS driven non-small-cell lung carcinoma (NSCLC) in mouse models, ablation of either ERK1 or ERK2 yielded only minimal elongation of lifespan, because of compensatory functions of these two kinases to maintain the ERK signaling pathway [15]. Complete ablation of ERK1/2 abolished tumor development, but resulted in rapid death of mice probably due to high toxicity, further supporting the high degree of functional similarity amongst these two isoforms.

Over the past decade, a number of protein methyltransferases have been identified and reported to methylate histone as well as non-histone proteins, enhancing cellular proliferation, thus playing a pivotal role in tumorigenesis [16]. ERK is known to have various post-translational modifications such as phosphorylation, acetylation, ubiquitilation and S-nitrosylation, however methylation of ERK has never been reported to date. In the present study, we first report that Suppressor of Variant 4–20 Homolog 1 (SUV420H1), a lysine methyltransferase known to methylate lysine 20 on histone H4, tri-methylates ERK1 at lysines 302 and 361. Our results imply that SUV420H1-mediated ERK1 methylation promotes tumorigenesis by enhancing ERK1 phosphorylation and transcription, resulting in activation of the ERK signaling cascade.

## RESULTS

### SUV420H1 methylates ERK1 at lysines 302 and 361 *in vitro*

**Figure 1:**
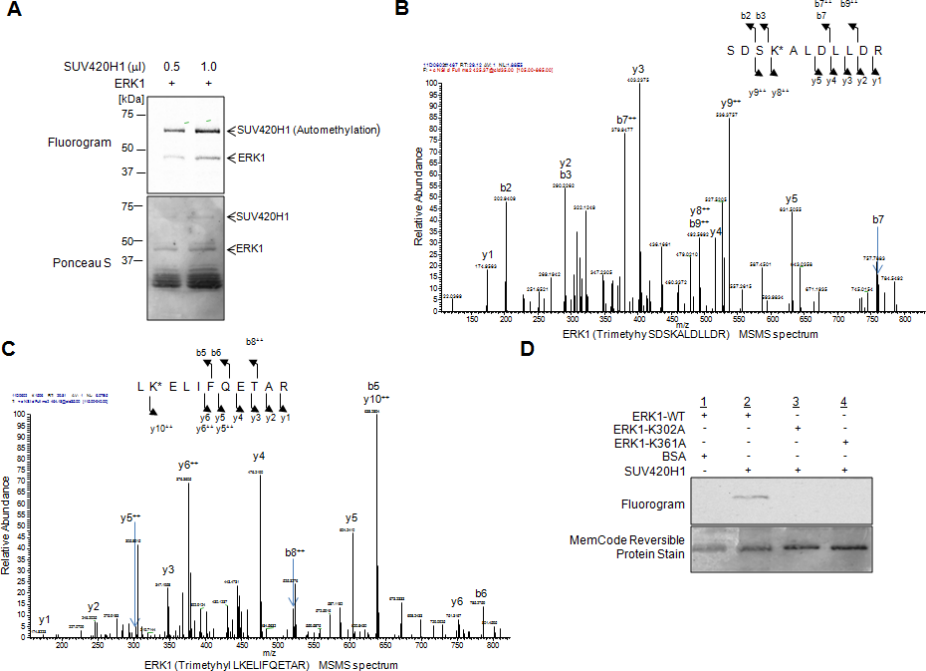
SUV420H1 methylates ERK1 *in vitro* (**A**) Recombinant ERK1 was methylated by SUV420H1 in a dose-dependent manner. Methylated ERK1 was detected by fluorography, and amounts of loading proteins were visualized by Ponceau S. (**B**, **C**) LC-MS/MS spectrum of the tri-methylated ERK1 peptide, including K302 (B, SDSKALDLLDR) and K361 (C, LKELIFQETAR). ERK1 recombinant protein reacted with SUV420H1 followed by SDS-PAGE. LC-MS/MS analysis was conducted after digestion of samples by trypsin. (**D**) *In vitro* methyltransferase assays using substituted ERK1 protein. Recombinant ERK1 protein was methylated by SUV420H1 *in vitro*, as visualized by fluorography. No methylation signals were detected for the K302-substituted ERK1 and K361-substituted ERK1 proteins. The amounts of loading proteins were validated and visualized by the MemCode^™^ Reversible Protein Stain (Thermo Fisher Scientific).

Aberrations in the ERK cascade are frequently observed in various cancer types, prompting us to study one of the terminal kinases of this hierarchical cascade. We performed an *in vitro* methyltransferase assay to identify potential enzymes that may methylate ERK1. Utilizing recombinant ERK1 protein and various recombinant histone methyltransferases likely to be involved in human tumorigenesis, we found SUV420H1 to methylate ERK1 (Supplementary Figure S1). To validate our findings, we incubated ERK1 with two different doses of SUV420H1 and observed that the ERK1 methylation signal increased in a dose-dependent manner (Figure 1A). Interestingly, we also observed methylation signals corresponding to SUV420H1 on the fluorogram, indicating its automethylation.

To identify the methylation site(s) of ERK1, we subsequently performed liquid chromatography-tandem mass spectrometry (LC-MS/MS) analysis and identified lysines 302 and 361 to be tri-methylated by SUV420H1 (Figure 1B and 1C). Next, we conducted an *in vitro* methyltransferase assay using wild-type ERK1 (ERK1-WT), K302-substituted ERK1 recombinant protein (ERK1-K302A) and K361-substituted ERK1 recombinant protein (ERK1-K361A) as substrates, SUV420H1 as the reacting enzyme and bovine serum albumin (BSA) as a negative control (Figure 1D). We detected ERK1 methylation in the presence of SUV420H1, while no methylation signal was seen in the ERK1-K302A and ERK1-K361A mutants, further supporting that SUV420H1 methylates ERK1 at these respective lysine residues *in vitro*.

### SUV420H1 is amplified in cancer and regulates the growth of cancer cells

**Figure 2:**
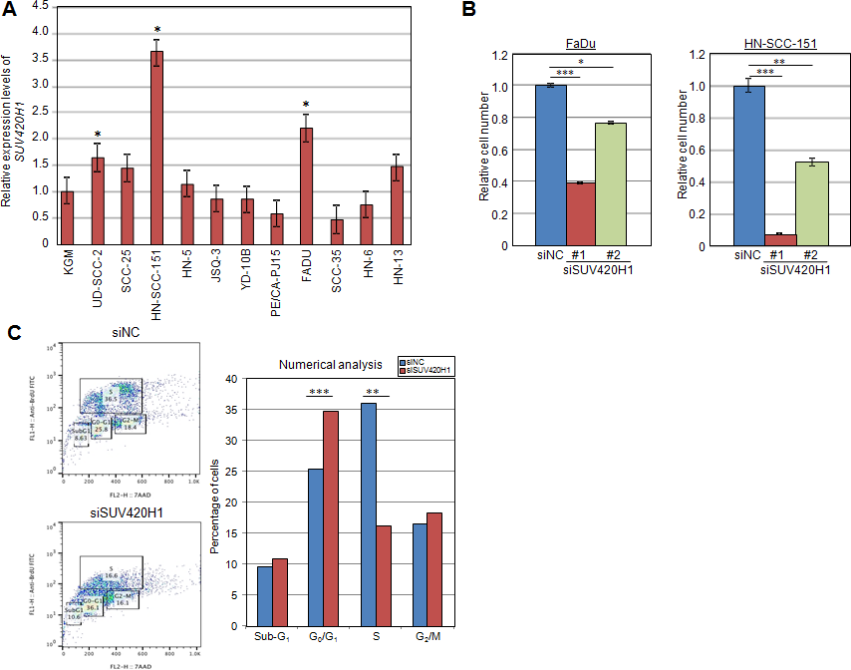
SUV420H1 is overexpressed in human cancer tissues (**A**) Quantitative real-time PCR of SUV420H1 in 11 SCCHN cell lines compared with normal control keratinocyte cell line (KGM). Three cell lines expressed significantly higher levels of *SUV420H1* mRNA compared to normal keratinocytes. mRNA levels were normalized by *GAPDH* and *SDH*. (**B**) MTT assays of FaDu and HN-SCC-151 SCCHN cells after treatment with siNC (control), siSUV420H1#1 and siSUV420H1#2. SUV420H1 knockdown suppressed cancer cell growth. Relative cell numbers are normalized to the number of siNC-treated cells (siNC = 1). Each condition was plated in quadruples, and *P* values were calculated using Student *t* test (*P < 0.05; **P < 0.01; ***P < 0.001). (**C**) Effect of siSUV420H1 on cell cycle kinetics in FaDu cells. Cell cycle distribution was analyzed by flow cytometry after coupled staining with fluorescein isothiocyanate (FITC)-conjugated anti-BrdU and 7-amino-actinomycin D (7-AAD) as described in Materials and Methods.

A search in the TCGA database showed that SUV420H1 is amplified in various types of human cancer, including breast, esophageal, bladder and head and neck cancers (Supplementary Figure S2). To further elucidate the importance of protein methyltransferase SUV420H1 in human cancer, we examined the expression profile of *SUV420H1* by quantitative real-time PCR (qRT-PCR) in 11 squamous cell carcinoma cell lines of the head and neck (SCCHN), and identified three SCCHN cell lines that expressed significantly higher levels of *SUV420H1* mRNA compared to normal keratinocytes (Figure 2A). To investigate whether SUV420H1 is essential for the growth of cancer cells, we carried out cell viability assays using two highly expressing *SUV420H1* SCCHN cell lines and one breast cancer cell line to examine a growth-suppressive effect. FaDu, HN-SCC-151 and MCF-7 cells were transfected with a control siRNA (siNC) or either of two SUV420H1-specific siRNAs (siSUV420H1 #1 and #2). We observed a significant growth-suppressive effect on the cells treated with SUV420H1 siRNAs compared with siNC using the Cell Counting Kit-8 system (Figure 2B and Supplementary Figure S3).

We then stained FaDu cells with Brd-U and 7-AAD to comprehensively evaluate the cell cycle status of cancer cells. Flow cytometric cell-cycle analysis revealed that cells transfected with *SUV420H1*-siRNA showed a significant reduction in the proportion of cells at the S phase, while that at the G_0_/G_1_ phase was significantly increased after knockdown of SUV420H1 (Figure 2C). These results imply that SUV420H1 is likely to promote G_1_ to S transition and enhance proliferation of SCCHN cells.

### Effect of SUV420H1-dependent methylation on the phosphorylation status of ERK1

**Figure 3:**
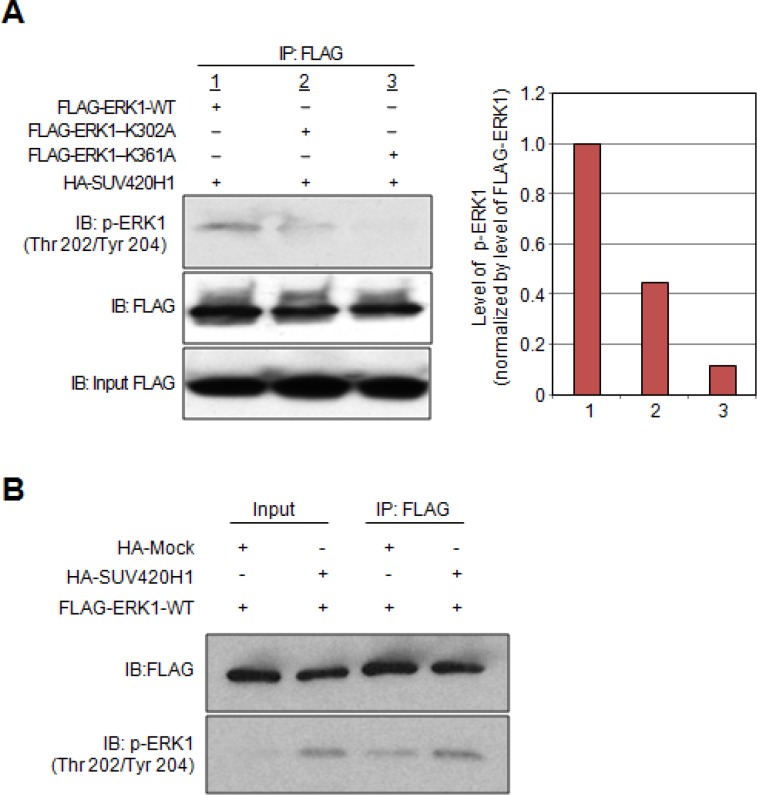
Effects of SUV420H1-dependent methylation on ERK1 activity (**A**) HeLa cells were co-transfected with HA-SUV420H1 and FLAG-ERK1-WT, or expression vectors containing the deletion variants (FLAG-ERK1-K302A, FLAG-ERK1-K361A). Cell lysates were immunoprecipitated with anti-FLAG M2 agarose beads. Samples were fractionated by SDS-PAGE and immunoblotted with anti-FLAG and anti-phospho ERK1 (Thr 202/Tyr 204) antibodies. Graphical representation of p-ERK1 levels normalized by FLAG-ERK1. (**B**) 293T cells were transfected with FLAG-ERK1-WT and HA-Mock or HA-SUV420H1. Cells were lysed with CelLytic^™^ M 48 hours after transfection, followed by immunoprecipitation using anti-FLAG M2 agarose. Samples were immunoblotted with anti-FLAG and anti-phospho ERK1 (Thr 202/Tyr 204) antibodies.

We previously reported that one of the molecular functions of protein lysine methylation is the enhancement of phosphorylation [16]. To address the biological importance of the individual K302 and K361 methylated lysine residues on the phosphorylation status of ERK1, we constructed expression vectors for FLAG-tagged lysine-substituted mutants. Subsequently, HeLa cells were transiently transfected with a plasmid expressing FLAG-tagged wild-type ERK1 (FLAG-ERK1-WT) or plasmids expressing either of the lysine-substituted mutants (FLAG-ERK1-K302A and FLAG-ERK1-K361A) in addition to HA-SUV420H1. We then immunoprecipitated FLAG-tagged proteins using whole cell lysates and carried out western blot analysis. Results showed reduction in the levels of p-ERK1 (Thr 202/Tyr 204) in the K302A-substituted ERK1 mutant and almost complete abolishment of p-ERK1 in the K361A-substituted ERK1 mutant (Figure 3A). These results support that methylation of K302 and K361 enhances ERK1 phosphorylation, and substitution of these lysine residues has a significant effect on ERK1 phosphorylation. We also examined the effect of SUV420H1 overexpression on the phosphorylation status of ERK1 in 293T cells. As shown in Figure 3B, we observed an increase of phosphorylation levels on ERK1 in the SUV420H1 overexpressing cells. Collectively, these results imply that SUV420H1-mediated K302/K361 ERK1 methylation enhances phosphorylation of ERK1.

### *In vivo* ERK1 methylation in cancer cells

**Figure 4:**
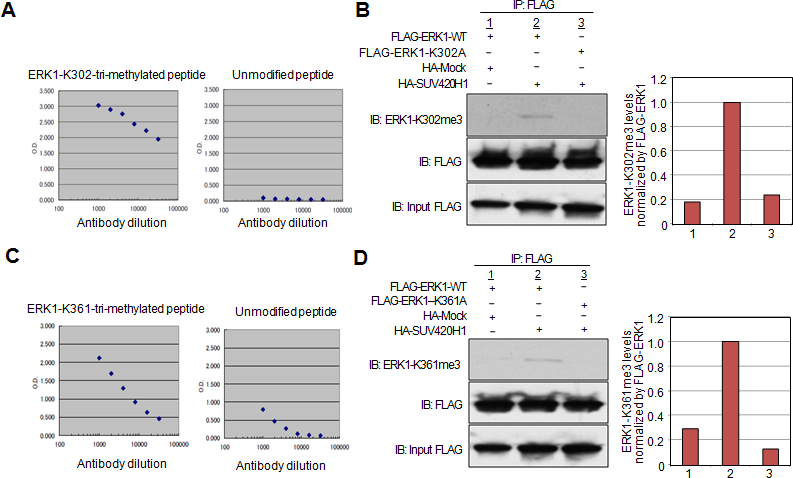
Confirmation of lysine 302 and lysine 361 methylation by specific antibodies *in vivo* (**A**) Determination of the titer and specificity of the anti-tri-methylated K302 ERK1 (Sigma-Aldrich) antibody analyzed by ELISA. (**B**) The FLAG-ERK1-WT or FLAG-ERK1-K302A vector was co-transfected with the HA-Mock or HA-SUV420H1 vector into HeLa cells. Whole cell lysates were immunoprecipitated with anti-FLAG M2 agarose beads. Immunoprecipitants were immunoblotted with anti-ERK1-K302me3 and anti-FLAG (Sigma-Aldrich) antibodies. Graphical representation of ERK1-K302me3 levels normalized by FLAG-ERK1. (**C**) Determination of the titer and specificity of the anti-tri-methylated K361 ERK1 (Sigma-Aldrich) antibody analyzed by ELISA. (**D**) The FLAG-ERK1-WT or FLAG-ERK1-K361A vector was co-transfected with the HA-Mock or HA-SUV420H1 vector into HeLa cells. Whole cell lysates were immunoprecipitated with anti-FLAG M2 agarose beads. Immunoprecipitants were immunoblotted with anti-ERK1-K361me3 and anti-FLAG (Sigma-Aldrich) antibodies. Graphical representation of ERK1-K361me3 levels normalized by FLAG-ERK1.

In order to clarify whether SUV420H1 methylates ERK1 *in vivo*, we generated antibodies recognizing the ERK1-K302-tri-methylated peptide or ERK1-K361-tri-methylated peptide. ELISA confirmed the specificity of the antibodies against either of the methylated peptides, which showed high affinity to the methylated ERK1 peptides, while not reacting with the unmodified peptides (Figure 4A and 4C). Subsequently, we co-transfected HeLa cells with FLAG-ERK1-WT or the substituted expression vectors, FLAG-ERK1-K302A or FLAG-ERK1-K361A, in the presence of HA-SUV420H1 or HA-Mock vector. FLAG-tagged proteins were then immunoprecipitated, and western blot analysis was performed to detect the methylated lysine residues of ERK1-WT. The ERK1 K302-methylation antibody bound specifically ERK1-WT *in vivo* as demonstrated by IP-WB analysis, while not being able to detect K302-substituted ERK1 (Figure 4B). Similar results were observed with the ERK1 K361-methylation antibody (Figure 4D). These findings validate that SUV420H1 methylates both lysines 302 and 361 *in vivo*, as demonstrated by these antibodies.

### SUV420H1 regulates ERK1 at the RNA level

**Figure 5:**
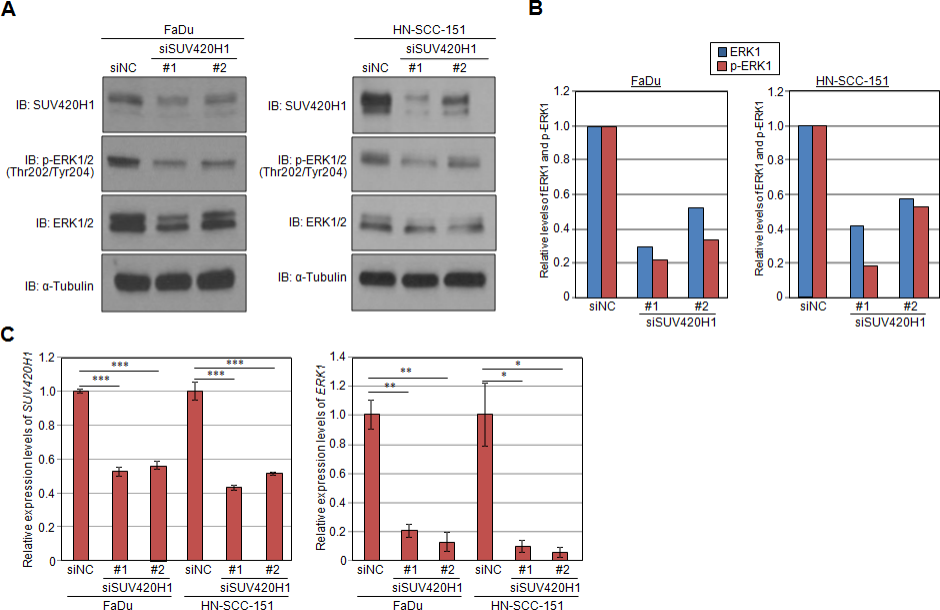
SUV420H1 knockdown attenuates levels of p-ERK1 and ERK1 expression (**A**) Levels of p-ERK1 and ERK1 were examined by western blot analysis 72 hours after SUV420H1 siRNA-mediated knockdown. SUV420H1 knockdown attenuated the phosphorylation levels of ERK1 and ERK1 protein. (**B**) Relative levels of p-ERK1 and ERK1 in siRNA-treated and control samples. Relative decrease in p-ERK1 levels was greater than the decrease in ERK1 protein levels. (**C**) *SUV420H1* and *ERK1* mRNA levels after SUV420H1 knockdown assessed by qRT-PCR. *P* values were calculated using Student *t* test (*P < 0.05; **P < 0.01; ***P < 0.001).

**Figure 6:**
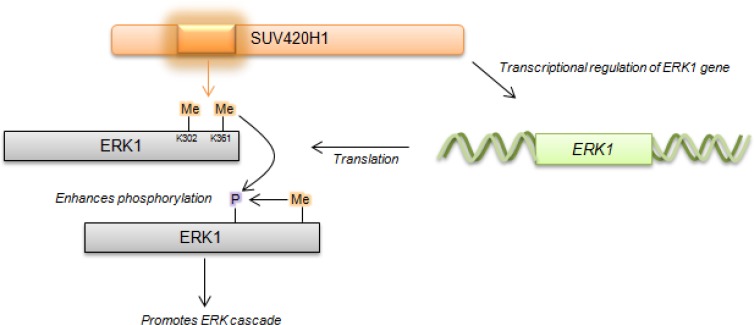
Proposed schematic of SUV420H1-mediated methylation on ERK1 activity SUV420H1-dependent methylation promotes ERK1 phosphorylation and impacts ERK1 expression levels via transcriptional regulation.

Given that ERK1 methylation enhances its phosphorylation, we examined the phosphorylation status of ERK1 following knockdown of SUV420H1. Knockdown of SUV420H1 significantly diminished the levels of p-ERK1, as visualized on western blot (Figure 5A). Moreover, knockdown of SUV420H1 significantly attenuated the levels of ERK1 protein, suggesting that SUV420H1 may also affect ERK1 protein expression levels. Quantification by densitometry (Figure 5B) suggested that the decrease of p-ERK1 levels may not only be attributed to the decrease of the total ERK1 protein levels, but also caused by downregulation of phosphorylation itself. To investigate whether *ERK1* is transcriptionally regulated by SUV420H1, we used quantitative real-time PCR to determine *ERK1* mRNA levels after SUV420H1 knockdown in FaDu and HN-SCC-151 cells. We confirmed knockdown of *SUV420H1*, as well as a decrease of *ERK1* mRNA levels in the siRNA-treated samples, supporting that ERK1 is also transcriptionally regulated by SUV420H1 (Figure 5C). Taken together, our findings suggest that SUV420H1 enhances ERK1 phosphorylation through the methylation of two lysine residues, and also regulates ERK1 transcription levels probably through histone modification (Figure 6).

## DISCUSSION

Lysine methylation of core histones plays a key role in regulating gene expression and impacting chromatin biology, and deregulation of histone lysine methylation has been linked to carcinogenesis, with many lysine methyltransferases reported to function as oncogenes [17–23]. In addition, accumulating evidence has unveiled that the function of protein lysine methyltransferases extends beyond that of histone methylation, as many protein methyltransferases have been reported to methylate non-histone protein substrates and play a critical role in human tumorigenesis [24–29]. Importantly, recent reports have identified several protein lysine methyltransferases as promising molecular targets for the development of novel anticancer therapy [16, 30–32].

In the current study, we demonstrated that lysines 302 and 361 of ERK1 are tri-methylated by protein methyltransferase SUV420H1, which is amplified in various types of cancer. siRNA-mediated knockdown of SUV420H1 significantly suppressed cancer cell growth through reduction of ERK1 phosphorylation as well as *ERK1* mRNA and protein expression. These findings imply that SUV420H1 appears to be an important target that can deregulate the ERK signaling cascade through lysine methylation of ERK in human cancer. Furthermore, the evidence presented here has clearly demonstrated that ERK1 methylation by SUV420H1 promotes ERK1 phosphorylation, and SUV420H1 is likely to enhance ERK1 transcription as well as protein levels, providing novel insights in the regulation of the ERK cascade. Since SUV420H1 has been reported to methylate histone H4 at lysine 20 (H4K20) [33–35], which is a marker of transcriptional repression, the transcriptional regulation of ERK1 by SUV420H1 seems to occur indirectly. Further analysis of ERK1 transcriptional regulation is warranted to delineate the mechanism of ERK1 expression by SUV420H1.

We previously reported that non-histone lysine methylation is an important regulator of gene expression and cellular signal transduction [16]. The interplay between methylation and phosphorylation of constituents of the ERK signaling cascade demonstrates the dynamics and impact of post-translational modifications on the regulatory function of the pathway. Mechanistically, methylation can promote or inhibit the progression of the ERK cascade by impacting the phosphorylation status of a given protein and/or its transcriptional regulation. Indeed, lysine and arginine methylations are known to have distinct functions in the fidelity of the pathway, as PRMT5-mediated RAF methylation at arginine 563 attenuates ERK1/2 phosphorylation [36], while SMYD3-mediated MAP3K2 methylation at lysine 260 augments ERK1/2 levels [37]. These findings imply that methylation of different amino acid residues has distinct biological functions on the ERK pathway.

In conclusion, SUV420H1-mediated ERK1 methylation promotes ERK1 phosphorylation and SUV420H1 may also regulate ERK1 expression levels at the transcriptional level, resulting in sustained ERK activation and cancer cell proliferation. Knockdown of SUV420H1 diminished ERK1 levels and attenuated the growth of cancer cells. Given that SUV420H1 is frequently overexpressed in various types of cancer and signaling molecules are attractive targets for the development of novel cancer therapeutics, inhibition of SUV420H1 may be a promising candidate for drug development.

## MATERIALS AND METHODS

### Antibodies

The following primary antibodies were used: anti-SUV420H1 (rabbit, Catalog No: 61415, 61416; Active Motif; dilution used in Western blotting: 1:2000), anti-Phospho-p44/42 MAPK (Erk1/2) (Thr 202/Tyr 204) (rabbit, D13.14.4E; Cell Signaling Technology; dilution used in WB: 1:2000), p44/42 MAPK (Erk1/2) (rabbit, 137F5; Cell Signaling Technology; dilution used in WB: 1:2000), anti-FLAG (mouse, M2; Sigma-Aldrich; dilution used in WB:1:2000), anti-human influenza hemagglutinin (rabbit, Y-11; Santa Cruz Biotechnology; dilution used in WB: 1:2000), anti–α-tubulin (mouse, DM1A; Calbiochem; dilution used in WB: 1:1000). An anti-K302 tri-methylated ERK1 antibody and anti-K361 tri-methylated ERK1 antibody (Sigma-Aldrich; dilution used in WB: 1:500) were produced in rabbit immunized with a synthetic peptide.

### Cell culture

The human breast cancer cell line MCF-7, the human embryonic kidney fibroblast cell line 293T and the human cervix carcinoma HeLa were obtained from American Type Culture Collection (ATCC), tested and authenticated by DNA profiling for polymorphic short tandem repeat (STR) markers (Supplementary Table S1). Squamous cell carcinoma cell lines HN-SCC-151 and FaDu were derived from patients with locoregionally advanced SCCHN and were kindly provided by Dr. Tanguy Seiwert (The University of Chicago). Detailed characteristics of each cell line are shown in Supplementary Table S2. All cell lines were grown in monolayers in appropriate media supplemented with 10% FBS and 1% antibiotic/antimycotic solution (Sigma-Aldrich): Dulbecco's Modified Eagle Medium (DMEM) for 293T and MCF-7; Dulbecco's Modified Eagle Medium: Nutrient Mixture F-12 (DMEM/F12) for HN-SCC-151; Minimal Essential Medium (MEM) for HeLa cells; Roswell Park Memorial Institute (RPMI) for FaDu; All cells were maintained at 37°C in humid air with 5% CO_2_ condition. Cells were transfected with FuGENE HD (Promega) according to the manufacturer's protocols.

### Cell cycle analysis

A 5-bromo-2′-deoxyuridine (BrdU) flow kit (BD Biosciences) was used to determine the cell cycle kinetics and to measure the incorporation of BrdU into DNA of proliferating cells [38–44]. The assay was performed according to the manufacturer's instructions. Briefly, FaDu cells were treated with siSUV420H1 or control siRNA (siNC), and cultured for 72 hours in a CO_2_ incubator at 37°C. Subsequently, cells were incubated with BrdU (final concentration 10 μM) for 1 hour. Floating and adherent cells were fixed in a solution containing paraformaldehyde and saponin, and incubated for 1 hour with DNAase at 37°C (30 μg per sample). Fluorescein isothiocyanate–conjugated anti-BrdU antibody (1:50 dilution in wash buffer; BD Pharmingen, San Diego, CA) was added and incubated for 20 minutes at room temperature. Cells were washed in wash buffer and total DNA was stained with 7-amino-actinomycin D (7-AAD; 20 μl per sample), followed by flow cytometric analysis using BDLSR II (BD Biosciences). Total DNA content (7-AAD) was determined by FlowJo software.

### *In vitro* methyltransferase assay

*In vitro* methyltransferase assays were described previously [45–49]. Briefly, recombinant SUV420H1 protein was incubated with recombinant ERK1 and 2 μCi S-adenosyl-L-[methyl-^3^H]-methionine (PerkinElmer) in a mixture of methylase activity buffer (50 mM Tris-HCl at pH 8.8, 10 mM DTT, and 10 mM MgCl_2_) for 1 hour at 30°C. After denaturing, samples were separated by sodium dodecyl sulfate – polyacrylamide gel electrophoresis (SDS-PAGE), blotted to polyviny- lidene difluoride membrane, and visualized by MemCode Reversible Stain (Thermo Fisher Scientific) and fluorography.

### Mass spectrometry

The reaction mixture of *in vitro* methyltransferase assay was analyzed by nano liquid chromatography–tandem mass spectrometry (LC-MS/MS) using LCQ Deca XP plus (Thermo Fisher Scientific). The peptides were separated using nano ESI spray column (100 μm [ID] × 50 mm [L]) packed with a reversed-phase material (Inertsil ODS-3, 3 μm; GL Sciences, Tokyo, Japan) at a flow rate 200 nl/min. The mass spectrometer was operated in the positive ion mode, and the spectra were acquired in a data-dependent MS/MS mode. The MS/MS spectra were searched against the in-house database using local MASCOT server (version 2.2.1; Matrix Sciences). The reaction mixture was desalted and applied to MALDI-TOF-MS using an Ultraflex (Bruker Daltonik GmbH).

### Quantitative real-time polymerase chain reaction

Specific primers for all human *GAPDH* (housekeeping gene), *SDH* (housekeeping gene) and *SUV420H1* were designed (detailed primer sequences in Supplementary Table S3). PCRs were performed using the ViiA^™^ 7 Real-Time PCR System (Thermo Fisher Scientific) following the manufacturer's protocol.

### Small interfering RNA transfection

Small interfering RNA (siRNA) oligonucleotide duplexes were purchased from Sigma-Aldrich for targeting SUV420H1 transcripts. siNegative control (siNC), which is a mixture of three different oligonucleotide duplexes, was used as control siRNAs. The siRNA sequences are described in Supplementary Table S4. siRNA duplexes (100 nM final concentration) were transfected using Lipofectamine RNAimax (Thermo Fisher Scientific) [50]. Cell viability was measured using the Cell Counting Kit-8 (Dojindo).

### RNA extraction

Total RNA was extracted using RNeasy Mini Kits (QIAGEN, Venlo, Netherlands) according to manufacturer's instructions. cDNA synthesis was performed using SuperScript^™^ III First-Strand Synthesis System for RT-PCR kit (Thermo Fisher Scientific). Detailed information for the primers is available in Supplementary Table S3.

### Western blot analysis

Whole-cell lysates were prepared from cells lysed with CelLytic M Lysis Reagent (Sigma-Aldrich) supplemented with complete protease inhibitor cocktail (Roche Applied Science) and phosphatase inhibitor. Whole-cell lysates or immunoprecipitated samples were separated by SDS-PAGE and blotted to nitrocellulose membrane. Protein bands were detected by incubating with HRP-conjugated antibodies (GE Healthcare) and visualized with enhanced chemiluminescence (GE Healthcare).

## SUPPLEMENTARY MATERIALS FIGURES AND TABLES


