# Supplementary material for: SUV420H1 enhances the phosphorylation and transcription of ERK1 in cancer cells
Source: Oncotarget. 2015 Nov 19;6(41):43162–71. doi: 10.18632/oncotarget.6351 (PMC4791223; doi:10.18632/oncotarget.6351)
Supplement: Supplementary file 1 [file oncotarget-06-43162-s001.pdf]

SUV420H1 enhances the phosphorylation and transcription of ERK1 in cancer cells

Supplementary Materials

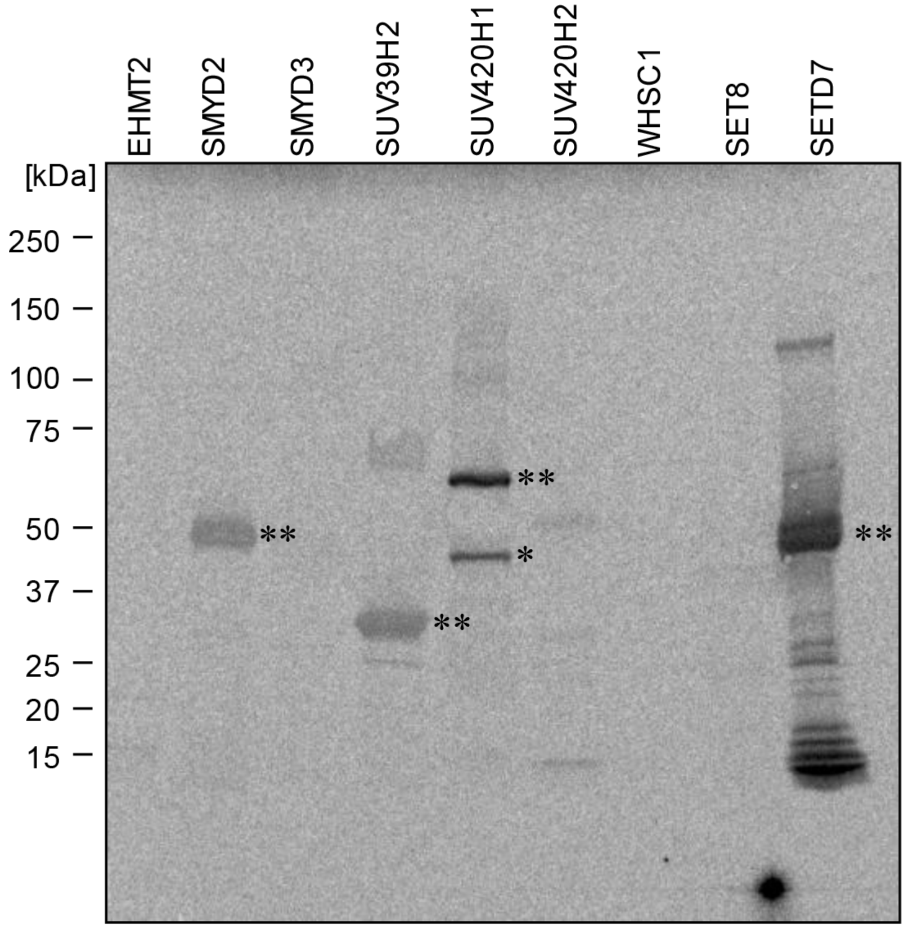

Supplementary Figure S1: SUV420H1 methylates ERK1 *in vitro*. Recombinant ERK1 protein was incubated with a variety of methyltransferases, and methylation signal was detected by autoradiography. *ERK1 methylation band. **automethylation band.

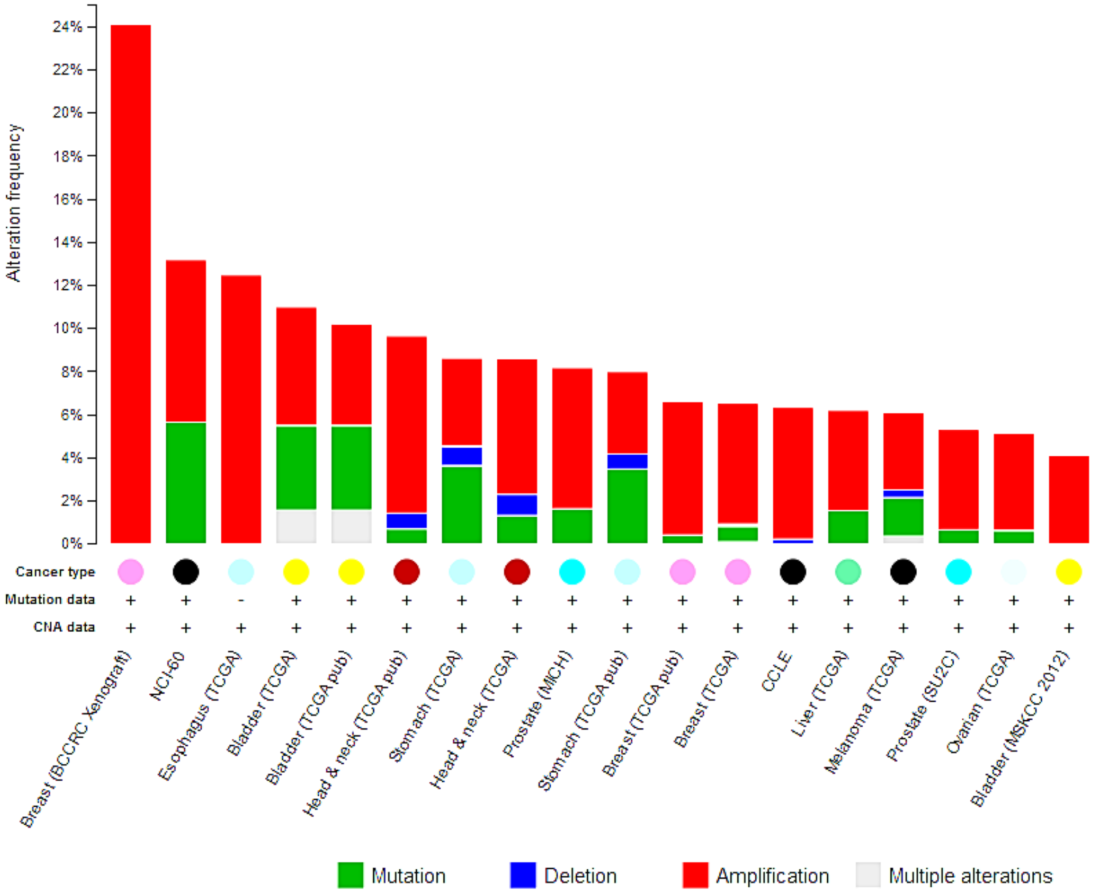

Supplementary Figure S2: Cross-cancer alteration summary for *SUV420H1*. The data were obtained from the TCGA database (http://www.cbioportal.org/public-portal/).

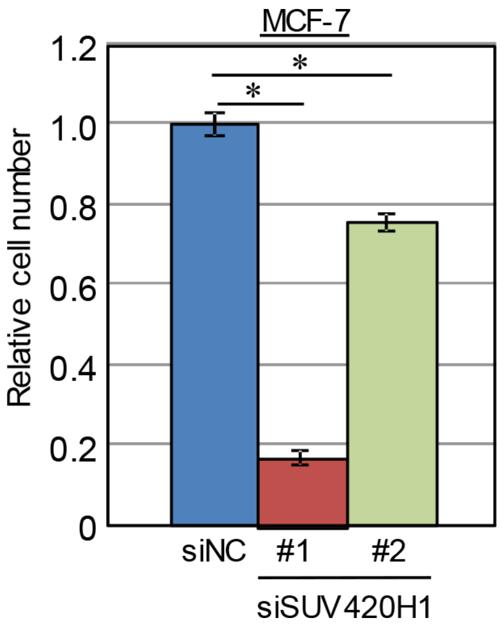

Supplementary Figure S3: Involvement of SUV420H1 in the growth of the breast cancer cell MCF-7. Relative cell number shows the value normalized to siNC-treated cells. Mean ± SD of four independent experiments. *P* values were calculated using Student's *t*-test (*$P < 0.05$).

Supplementary Table S1: Information of certificated cell lines

Name	Origin	Certification institution	Tested method	DNA profile or characteristics
MCF7	human breast cancer	ATCC	STR	Amelogenin: X/X CSF1PO: 10/10 D13S317: 11/11 D16S539: 11/12 D5S818: 12/12 D7S820: 8/9 THO1: 6/6 TPOX: 9/12 vWA: 14/15 D3S1358: 16/16 D21S11: 30/30 D18S51: 14/14 Penta E: 7/12 Penta D: 12/12 D8S1179: 10/14 FGA: 23/25 CLS 2011.
293T	human embryonic kidney fibroblast	ATCC	STR	Amelogenin: X CSF1PO: 11, 12 D13S317: 12, 14 D16S539: 9, 13 D5S818: 8, 9 D7S820: 11 THO1: 7, 9.3 TPOX: 11 vWA: 16, 18, 19
HeLa	human cervix carcinoma	ATCC	STR	Amelogenin: X, Y CSF1PO: 11, 12 D13S317: 11, 14 D16S539: 9, 11 D5S818: 11, 12 D7S820: 10, 11 THO1: 8 TPOX: 8 vWA: 15

ATCC; American Type Culture Collection

Supplementary Table S2: Characteristics of SCCHN cell lines

Cell name	TNM stage	Specimen site	Gender	HPV status
HN-SCC-151	T3N0M0	Oral tongue	−	HPV16-negative
FaDu	−	Hypopharynx	Male	HPV16-negative

Supplementary Table S3: Primer sequences for quantitative RT-PCR

Gene name	Primer sequence
GAPDH (housekeeping gene)-f	5′ GCAAATTCCATGGCACCGTC 3′
GAPDH (housekeeping gene)-r	5′ TCGCCCCACTTGATTTTGG 3′
SDH (housekeeping gene)-f	5′ TGGGAACAAGAGGGCATCTG 3′
SDH (housekeeping gene)-r	5′ CCACCACTGCATCAAATTCATG 3′
SUV420H1-f	5′ AGAAATCATTGCAAGCGGCTGGAG 3′
SUV420H1-r	5′ CTGGCTCCTTATCTTTTTTAATGG 3′

Supplementary Table S4: siRNA sequences

siRNA name		Sequence
	Target#1	Sense: 5′ AUCCGCGCGAUAGUACGUA 3′
siNegative control		Antisense: 5′ UACGUACUAUCGCGCGGAU 3′
(Cocktail)	Target#2	Sense: 5′ UUACGCGUAGCGUAAUACG 3′
		Antisense: 5′ CGUAUUACGCUACGCGUAA 3′
	Target#3	Sense: 5′ UAUUCGCGCGUAUAGCGGU 3′
		Antisense: 5′ ACCGCUAUACGCGCGAAUA 3′
siSUV420H1#1		Sense: 5′ CUGCGUUUAUAAACCAUGA 3′
		Antisense: 5′ UCAUGGUUUAUAAACGCAG 3′
siSUV420H1#2		Sense: 5′ GUGUCAACUGGUCGAGAUA 3′
		Antisense: 5′ UAUCUCGACCAGUUGACAC 3′
